# Changes in patient profiles in the cardiology department of the University Hospital Gabriel Touré (UH-GT) : results of two cross-sectional studies of 2010 and 2022

**DOI:** 10.1186/s12872-024-03890-3

**Published:** 2024-06-01

**Authors:** Hamidou Oumar BA, Y Camara, M Poudiougou, I Sangaré, N Sidibé, D Traoré, I Menta

**Affiliations:** 1 University Hospital “Gabriel Touré”, Bamako, Mali; 2 University Hospital “Sidy Bocar Sall”, Kati, Mali; 3 University Hospital “Point G”, Bamako, Mali

**Keywords:** Cardiovascular risk factor, Trend, Cardiology, Mali, University hospital, Outpatient

## Abstract

**Background:**

Little is known about patient profile changes in medical facilities in our country, leading to this study to describe and compare patient profiles in 2010 and 2022.

**Patients and methods:**

This was a cross-sectional study with new outpatients aged 15 years and more seen in the cardiology department of the UH-GT. Measurements included height, weight and body mass index (BMI). Systolic blood pressure (SBP) and diastolic blood pressure (DBP) were recorded. Quantitative data are presented as the mean with standard deviation, and categorical one as proportions. Statistical tests were the t test to compare means and chi-test for categorical variables. The level of significance was set to 0.05.

**Results:**

The sample consisted of 515 new patients (199 in 2010 and 316 in 2022) with 59.1% female in 2010 and 60.1% in 2022 (*p* = 0.821). We noticed an increase in hypertension (59.1–71.8%, *p* = 0.003) and a decrease in tobacco smoking (from 13 to 05.4%, *p* = 0.002) and stroke (from 05.8 to 02.2%, *p* = 0.033). Height increased significantly from 1.59 m to 1.66 m, *p* = 0.002. SBP and DBP showed significant decreases in their means from 155.43 to 144.97 mmHg, p = < 0.001 for SBP and from 95.53 to 89.02 mmHg, p = < 0.001 for DBP.

**Conclusions:**

Cardiovascular risk factors showed different trends with decreasing tobacco smoking, similar to systolic and diastolic blood pressure, albeit with an increase in hypertension prevalence. Other CVrf values increased. Awareness campaigns must be reinforced and maintained to obtain their decrease.

## Background

Cardiology is one of the solicited departments in medical facilities due to different factors. Cardiovascular diseases (CVDs) are the leading cause of death according to the WHO [1]. Another fact is that the burden of CVD with prevalent cases nearly doubled from 271 in 1990 to 523 million in 2019 [2] and that over 70% of CVD cases and deaths in the overall cohort were attributed to modifiable risk factors [3]. According to Hinton, CVD represents one-fifth of adult patients [4], and continent-wide, the median age-standardized incidence was lower in middle-income countries than in high-income countries (1039 vs. 1224) (IQR 11,061,356)] for both females and males [5].

Low- and middle-income countries (LMICs) are most affected, with more than 80% of these cases [6, 7] and more than doubling in our neighboring countries [8].

However, little is known about patient profile changes in medical facilities in our country, leading to this study, which aims to describe and compare patient profiles over a 12-year time interval (2010 and 2022).

## Patients and methods

### Study design

The study was designed as a cross-sectional study including all new outpatients aged 15 years and more seen in the cardiology department of the UH-GT.

We replicated a previous study performed from December ^1,^ 2009, to January 31, 2010, in the cardiology department of the University Hospital Gabriel Touré in Bamako (UH-GT). The actual study was conducted from July 04 to December 02, 2022.

### Setting

The UH-GT is an university teaching hospital with a cardiology department in Bamako, Mali. In the setting are relevant laboratory tests available as well as imaging modalities such as transthoracic echocardiography, electrocardiography and long time blood pressure and heart rhythm recording systems.

### Participants

Include were all participants aged 15 years and more, regardless of sex and seen in the outpatient unit in the study period.

Informed consent was obtained freely and voluntarily after being explanation of the study goal, collected data, the confidential treatment of their data and more importantly that the participation is not mandatory. We didn’t need to exclude patients.

### Variables and data Collection

Study variables involved sociodemographics (age, gender, residence and education level) obtained through direct interrogation of patients. Education level was evaluated as school attendance in years and presented as categories with none, primary, secondary and university for 0, 1–9, 10–12 and more than 12 years of school attendance, respectively.

Measurements including height in m and weight in kg were recorded as quantitative variables. Body mass index (BMI) in ^kg/m2^ was calculated from height and weight as weight/height^2^. BMI was then classified as normal, overweight and obese for 18,5- less than 25, ≥ 25 and < 30 and ≥ 30, respectively.

Systolic blood pressure (SBP) and diastolic blood pressure (DBP) were recorded as quantitative variables in mmHg after being measured 3 consecutive times using an automatic sphygmomanometer (OMRON) after a 5-minute rest.

Data were collected through a validated survey formular and added to the same Microsoft Access database from the 2010 study.

Analysis was performed using IBM SPSS software. Quantitative data are presented as the mean with standard deviation, and categorical data are presented as proportions. Statistical tests were the t test to compare means and chi-test for categorical variables. The level of significance was set to 0.05.

## Results

The sample consisted of 515 new patients (199 in 2010 and 316 in 2022).

The female sex was most represented (59.1%) in 2010 and 60.1% in 2022, with a p value of 0.821. There were more patients with increasing age, but the differences were not significant. The distribution of school attendance showed no significant difference (Table 1).

**Table 1:** distribution of the sociodemographic characteristics of 515 patients according to the year of study

Variables		2010 N(%)	2022 N(%)	p
Sex	Male	085 (40.9)	126 (39.9)	0.821
	Female	123 (59.1)	190 (60.1)	
Age group (years)	< 30	033 (15.9)	038 (12.0)	0.392
	40–44	037 (17.8)	072 (22.8)	
	45–59	062 (29.8)	089 (28.2)	
	≥ 60	076 (36.5)	117 (37.0)	
School attending	None	114 (58.7)	161 (52.3)	0.516
	Primary	043 (21.9)	082 (26.6)	
	Secondary	026 (13.3)	042 (13.6)	
	University	012 (06.1)	023 (07.5)	

The distribution of cardiovascular risk factors (CVrf) showed an increase in hypertension (59.1–71.8%, *p* = 0.003) and a decrease in tobacco smoking (from 13 to 05.4%, *p* = 0.002). A similar decrease in stroke was found (from 05.8 to 02.2%, *p* = 0.033). Diabetes, alcohol consumption and obesity did not show a significant difference (Table 2).

**Table 2:** Distribution of cardiovascular risk factors among 515 newly diagnosed hypertensive patients according to year of study

Variables		2010	2022	N	p
Hypertension		123 (59.1)	227 (71.8)	350	0.003
Diabetes		013 (06.2)	026 (08.2)	039	0.399
Tobacco smoking		027 (13.0)	017 (05.4)	044	0.002
Alcohol consumption		204 (01.9)	312 (01.3)	008	0.548
Stroke		012 (05.8)	007 (02.2)	019	0.033
Body mass index (kg/m^2^)	< 25	106 (57.0)	164 (52.4)	270	0.423
	25–29	044 (23.7)	073 (23.3)	117	
	≥ 30	036 (19.4)	076 (24.3)	112	

In terms of family history, only hypertension and diabetes were found to be different. A family history of hypertension decreased from 51.9 to 17.4%, *p* < 0.001, and diabetes decreased from 10.6 to 04.4%, *p* = 0.006 (Table 3).

**Table 3:** distribution of family cardiovascular risk factors and death according to study year

Variables		2010	2022	p
Hypertension		108 (51.9)	055 (17.4)	< 0.001
Diabetes		022 (10.6)	014 (04.4)	0.006
Stroke		005 (02.4)	002 (00.6)	0.084
Death due to	Hypertension	004 (01.9)	004 (01.3)	0.548
	Diabetes	001 (00.5)	001 (00.3)	0.765

Among anthropometric variables, only mean height increased significantly from 1.59 to 1.66%, *p* = 0.002. Age, weight and body mass index (BMI) showed increases that were not significant (Table 4).

**Table 4:** distribution of mean anthropometric variables according to study year for 199 2010 patients 2010 and 316 2022 patients

Variables	t(df)	2010		2022		Diff*	p
		Mean	SD	Mean	SD		
Age (years)	-0.190(522)	50.12	18.635	50.41	16.281	-0.293	0.849
Weight (kg)	-0.408(502)	70.77	17.457	71.43	17.929	-0.668	0.683
Height (m)	-3.113(512)	1.59	0.393	1.66	0.094	-0.072	0.002
BMI^+^ (kg/m^2^)	-1.746(501)	24.99	5.983	25.996	6.430	-1.009	0.081

*Difference^+^Body mass index

Systolic blood pressure (SBP) and diastolic blood pressure (DBP) showed significant decreases in their means. Therefore, SBP decreased from 155.43 to 144.97 mmHg, p = < 0.001, and DBP decreased from 95.53 to 89.02 mmHg, p = < 0.001, giving mean differences of 10.459 and 6.509, respectively. The mean difference in heart rate was not significant (*p* = 0.144) (Table 5).

**Table 5:** distribution of mean pressure values according to study year for 199 2010 patients 2010 and 316 2022 patients

Variables	t(df)	2010		2022		Diff*	p
		Mean	SD	Mean	SD		
SBP^+^ (mmHg)	3,841(513)	155.43	33,070	144.97	28,053	10.459	< 0.001
DBP^++^ (mmHg)	3,975(513)	95.53	18.1891	89.02	18.030	6.509	< 0.001
HR^+++^ (/min)	1.465(508)	86.93	17.202	84.66	16.884	2.272	0.144

*Difference^+^systolic blood pressure ^++^diastolic blood pressure ^+++^ heart rate

## Discussion

There is a lack of trend studies in our environment, and this study is the first in its kind in our department.

The main findings resulting from our study are as follows:

- Patients attending the facility.
  The patients of the two independent samples composing this study were similar in terms of gender, age and education level, as shown in Table I, meaning that no structural change occurred.
  The prevalence of females is a common finding in most studies [8–11], but for some authors, it warrants periodic re-examination [12]. Many factors, including psychosocial problems or distress and easier divulgation of personal information [13], have been underscored. This higher rate of female attendance and a female excess of physical symptoms has been found to be independent of the number and nature of symptoms measured, the time frame involved, and the response format and scoring method used [14].
- **Distribution of cardiovascular risk factors**.
  We found a significant increase in hypertension in this study. There is no clear trend in the distribution of CVrf worldwide. Most authors found an increase in hypertension and dyslipidemia. So found Jardim et al. an increase of hypertension prevalence from 6.0 to 16.7% with *p* = 0.024 and dyslipidemia from 4.0 to 19.14% with *p* = 0.002) [15]. This increase was similar to Fores data with 15.4% [16] and more pronounced particularly for sub-Saharan Africa about 20% [17] [15–17]. There is even an increase in the mean number of CVrf in men and women [18]. Data from the Czech Republic found a decrease in total cholesterol and the mean population blood pressure [19], whereas Castel-Faced found no significant changes in CVrf in a cluster analysis [20].
  Diabetes as a major CVrf shows a decrease in high-income countries and an increase in low- and middle-income countries [21, 22]. Recent studies show a shifting risk factor profile toward a younger population with lower rates of established CVD from the onset of the COVID-19 pandemic [23].
  Tobacco smoking, as another major CVrf, decreased in this study, consistent with most studies [19], but data from Switzerland pointed out a striking increase in tobacco smoking in women [18]. The different regular awareness campaigns from the state and stakeholders such as the Malian cardiac society, among others, are possibly playing a role in this decrease.
- **Anthropometric variables**.
  BMI in this study shows an increase that was not significant and not consistent with literature data showing an increase in BMI events for children and adolescents [24], and with continuing trends, by 2025, global obesity prevalence will reach 18% in men and surpass 21% in women; severe obesity will surpass 6% in men and 9% in women [25, 26].
- **Blood pressure on admission**.
  We found a decrease in the levels of SBP and DBP on admission, although hypertension increased. This is in opposition to studies with rising SBP and DBP, as reported by Churilova [27], and the relationship of mean blood pressure and the prevalence of hypertension [28]. An explanation could be the role of traditional medication that is not always confessed and therefore not recorded during patient questioning.

### Limitations

Despite its strength through providing data on changes in patient profiles in the cardiology department in a low-income country, this study has some limitations. It was conducted without a funding not allowing to include more patients and extend to several months. The lack of labor data essentially due to their cost for patients (most of them don’t benefit from a medical insurance) is another limitation of this study.

## Conclusion

Cardiovascular risk factors showed different trends with decreasing tobacco smoking, similar to systolic and diastolic blood pressure, albeit with an increase in hypertension prevalence. Other CVrf values increased. Awareness campaigns must be reinforced and maintained to obtain a decrease in all CVrf.

## Data Availability

The data used to support the findings of this study are available from the corresponding author upon request.

## Abbreviations

BMI: body mass index
CVD: cardiovascular diseases
CVrf: cardiovascular risk factors
DBP: diastolic blood pressure
HR: health rate
IBM SPSS: International Business Machine Statistical Software Package for Social Sciences
LMIC: low- and middle-income countries
SBP: systolic blood pressure
UH-GT: University Hospital Gabriel Touré
WHO: World Health Organization
